# Cerebrospinal fluid levels of glial marker YKL-40 strongly associated with axonal injury in HIV infection

**DOI:** 10.1186/s12974-019-1404-9

**Published:** 2019-01-24

**Authors:** Linn Hermansson, Aylin Yilmaz, Markus Axelsson, Kaj Blennow, Dietmar Fuchs, Lars Hagberg, Jan Lycke, Henrik Zetterberg, Magnus Gisslén

**Affiliations:** 1 Department of Infectious Diseases, University of Gothenburg, Gothenburg, Sweden; 2 Department of Neurology, University of Gothenburg, Gothenburg, Sweden; 3 Department of Psychiatry and Neurochemistry, Sahlgrenska Academy, University of Gothenburg, Gothenburg, Sweden; 4 Clinical Neurochemistry Lab, Sahlgrenska University Hospital, Mölndal, Sweden; 5 Division of Biological Chemistry, Biocenter, Innsbruck Medical University, Innsbruck, Austria; 6 Institute of Neurology, University College London, London, UK

**Keywords:** HIV, Cerebrospinal fluid, YKL-40, Neurofilament protein

## Abstract

**Background:**

HIV-1 infects the central nervous system (CNS) shortly after transmission. This leads to a chronic intrathecal immune activation. YKL-40, a biomarker that mainly reflects activation of astroglial cells, has not been thoroughly investigated in relation to HIV. The objective of our study was to characterize cerebrospinal fluid (CSF) YKL-40 in chronic HIV infection, with and without antiretroviral treatment (ART).

**Methods:**

YKL-40, neopterin, and the axonal marker neurofilament light protein (NFL) were analyzed with ELISA in archived CSF samples from 120 HIV-infected individuals (85 untreated neuroasymptomatic patients, 7 with HIV-associated dementia, and 28 on effective ART) and 39 HIV-negative controls.

**Results:**

CSF YKL-40 was significantly higher in patients with HIV-associated dementia compared to all other groups. It was also higher in untreated neuroasymptomatic individuals with CD4 cell count < 350 compared to controls. Significant correlations were found between CSF YKL-40 and age (*r* = 0.38, *p* < 0.001), CD4 (*r* = − 0.36, *p* < 0.001), plasma HIV RNA (*r* = 0.35, *p* < 0.001), CSF HIV RNA (*r* = 0.35, *p* < 0.001), CSF neopterin (*r* = 0.40, *p* < 0.001), albumin ratio (*r* = 0.44, *p* < 0.001), and CSF NFL (*r* = 0.71, *p* < 0.001). Age, CD4 cell count, albumin ratio, and CSF HIV RNA were found as independent predictors of CSF YKL-40 concentrations in multivariable analysis. In addition, CSF YKL-40 was revealed as a strong independent predictor of CSF NFL together with age, CSF neopterin, and CD4 cell count.

**Conclusions:**

CSF YKL-40 is a promising biomarker candidate for understanding the pathogenesis of HIV in the CNS. The strong correlation between CSF YKL-40 and NFL suggests a pathogenic association between astroglial activation and axonal injury, and implies its utility in assessing the prognostic value of YKL-40.

## Background

Like other lentiviruses, HIV-1 (henceforth HIV) is a neurotropic virus. It enters the central nervous system (CNS) early after transmission and persists throughout the course of infection [1]. After traversing the blood-brain barrier (BBB), HIV productively infects perivascular macrophages and microglia, leading to an inflammatory response with the activation of these cells [2]. The infected cells produce viral particles and release viral proteins and cellular products such as cytokines, quinolinic and arachidonic acid, platelet-activating factor, and nitric oxide, all of which are toxic against neurons and astrocytes [3]. Astrocytes can also be infected, although to a lesser extent than monocytic cells, and probably not productively, but they can be activated by microglia [4, 5]. Proliferation of astrocytes (astrogliosis) is one of the neuropathological hallmarks of HIV-associated dementia (HAD). Astrocyte activation is also linked to increased permeability of the BBB, which enhances the leakage of cells and serum products into the CNS [6]. HIV does not productively infect neurons, but an intrathecal inflammation is associated with synaptodendritic injury, neuronal dysfunction, and apoptosis [7]. Although the exact pathogenesis remains uncertain, previous studies have indicated that the above inflammation is mediated by infected macrophages and microglia interacting with astrocytes [8]. This immune activation, perhaps in combination with toxic metabolites from immune cells and viral proteins, may be the driver of neuronal injury in HIV infection [9].

Before effective antiretroviral treatment (ART) was available, up to 30% of people living with HIV (PLHIV) developed HAD with cognitive and motor impairment [3]. The prevalence of HAD dramatically decreased after the introduction of combination ART. However, despite effective ART, milder forms of HIV-associated neurocognitive disorders (HAND) are still frequently reported among PLHIV, impacting neurocognitive function and quality of life [10, 11].

Neopterin is a marker of intrathecal immune activation that has been thoroughly studied in relation to HIV [12–16]. It is mainly produced by macrophages and related cells, such as dendritic cells, after stimulation by interferon gamma (IFN-γ) and tumor necrosis factor alpha (TNFα) from activated T cells [12, 13]. Cerebrospinal fluid (CSF) concentrations of neopterin increase in all stages of untreated HIV infection, with the highest levels seen in patients with CNS opportunistic infections and HAD [14]. Neopterin levels decrease substantially after treatment initiation but remain above normal reference levels in approximately 40% of PLHIV with effective ART [15, 16]. A correlation between neopterin and axonal injury, as measured by CSF neurofilament light protein (NFL), has been found, supporting the hypothesis of neuroinflammation as a cause of CNS injury in PLHIV [16].

YKL-40 (chitinase-3-like protein 1 [CHI3L1]) is a glycoprotein expressed by inflammatory cells during differentiation [17]. Elevated serum levels of YKL-40 have been found in several inflammatory diseases and malignancies [17–20]. The physiological role of YKL-40 is unclear, but it is believed to be involved in tissue remodeling in inflammation [21]. YKL-40 levels in CSF are elevated particularly in neurodegenerative and neuroinflammatory diseases such as Alzheimer’s disease [22] and multiple sclerosis (MS) [23, 24]. While expressed by microglia and macrophages in vitro, YKL-40 has been seen to accumulate around activated astrocytes in vivo [25, 26]. The expression of YKL-40 in neuroinflammation appears to be related primarily to astrocyte activation induced by interleukin 1 beta (IL-1β) and TNFα released by macrophages [26, 27]; however, the exact contribution from different cell types has not been fully elucidated.

Increased levels of CSF YKL-40, but not plasma YKL-40, have previously been found in macaques with simian immunodeficiency virus (SIV) 2–8 weeks before development of encephalitis [28]. In humans, initiation of ART was associated with lower CSF YKL-40 concentrations in acute, but not in chronic, HIV. This could indicate that it is possible to prevent or decrease intrathecal immune activation by early initiation of ART [29].

The objective of this study was to determine CSF YKL-40 in different stages of chronic HIV infection, with and without ART, and to investigate how it relates to axonal injury, as measured by CSF NFL, for better understanding of the HIV neuropathogenesis.

## Methods

### Patients

In this retrospective, cross-sectional study, we analyzed archived CSF samples from 120 PLHIV followed at the Department of Infectious Diseases, Sahlgrenska University Hospital, Gothenburg, Sweden, and from 39 HIV-negative controls. The HIV-infected individuals were divided into three groups: (1) neuroasymptomatic (NA) without ART (*n* = 85), further stratified into four categories according to CD4+ T cell count; (2) HAD, without ART (*n* = 7); and (3) patients on suppressive ART (*n* = 28). The diagnosis of HAD was based on the Centers for Disease Control and Prevention and American Academy of Neurology task force criteria using standard laboratory, neuropsychological testing, and clinical evaluations [30, 31]. Patient characteristics are presented in Table 1. Controls were healthy volunteers (69% men) with a median age of 40 years (Table 1)

**Table 1:**
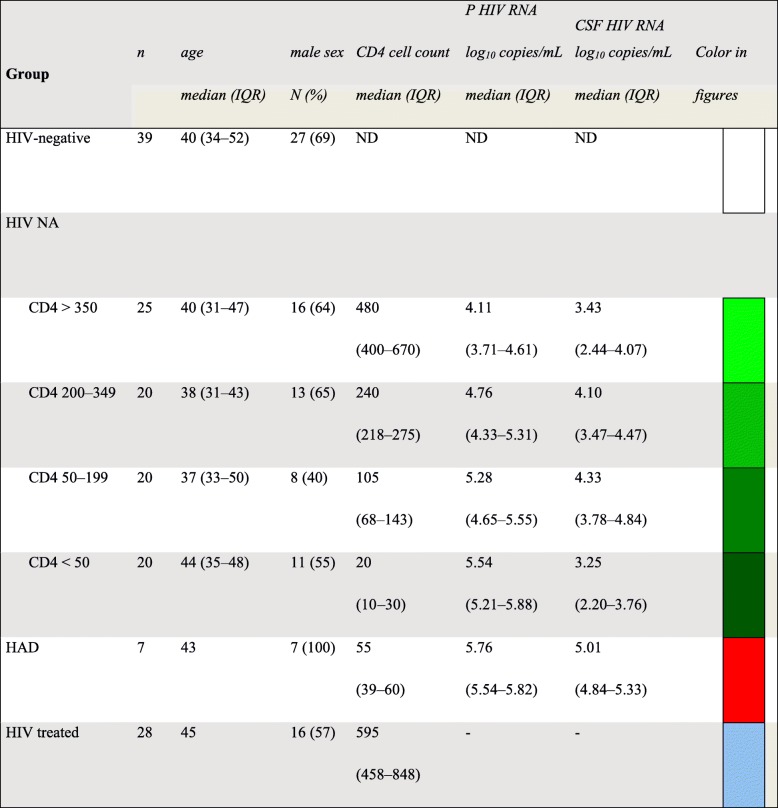
Group characteristics

*NA* neuroasymptomatic, *HAD* HIV-associated dementia, *IQR* interquartile range

### Laboratory analysis

Low-speed centrifugation was used to remove cells from CSF and blood samples. They were then aliquoted and frozen to − 70 °C within 1 h of obtaining the samples by lumbar puncture and stored until analysis.

CSF and serum YKL-40 levels were measured by solid-phase sandwich ELISA (R&D Systems, Minneapolis, MN, USA) with a lower detection limit of 2.32 pg/mL.

CSF NFL was measured using a sandwich ELISA method (NF-light® ELISA kit, UmanDiagnostics, AB, Umeå, Sweden). The lower limit of quantification was 50 ng/L. Upper reference values are age dependent as described by Yilmaz et al. [32].

HIV RNA in CSF and plasma was measured using the Roche Amplicor Monitor version 1.5 or Roche TaqMan assay version 1 or 2 with lower quantification limits of 50 and 20 copies/mL, respectively (Hoffman La-Roche, Basel, Switzerland).

Neopterin was analyzed in CSF and serum using a commercially available immunoassay (BRAHMS, Berlin, Germany) with an upper normal limit of 5.8 nmol/L in CSF and 8.8 nmol/L in serum [14, 15].

Other measurements, including blood CD4+ T cell counts, CSF white blood cells (WBC), and CSF and blood albumin, were performed in the local clinical laboratory using standard clinical chemistry assays. Albumin ratio was calculated as CSF albumin (mg/L)/plasma albumin (g/L) and used to evaluate BBB function. Reference values were < 6.8 for individuals younger than 45 years and < 10.2 for individuals 45 years and older.

### Statistical analysis

Descriptive statistics were performed using SPSS software (IBM SPSS version 24) or Prism (GraphPad software version 7.0). Continuous variables, except age and CSF leukocyte count, were log_10_ transformed. To compare continuous variables between multiple groups, one-way analysis of variance with Tukey’s multiple comparison was used. Correlations were analyzed with Pearson correlation coefficients. Predictors of YKL-40 and CSF NFL were analyzed by multiple linear regression analysis with forward selection.

## Results

**Fig. 1:**
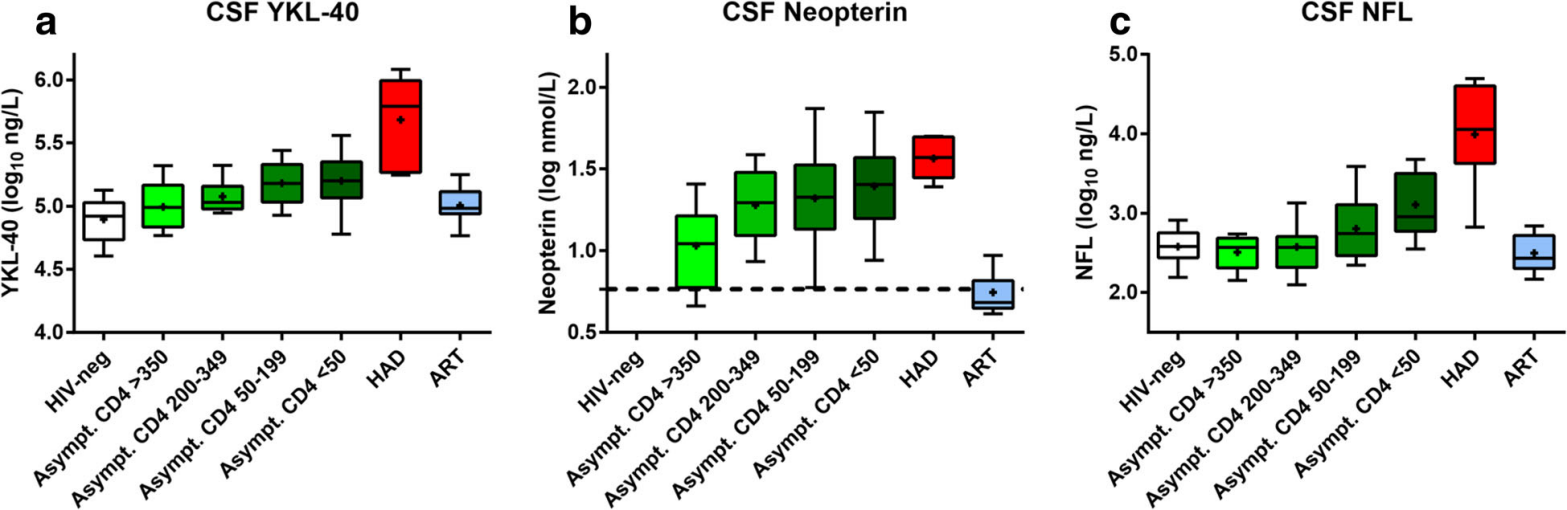
Group comparisons of CSF (cerebrospinal fluid) levels of **a** YKL-40, **b** neopterin, and **c** NFL. Boxes in panels depict median and IQR, whiskers show 10–90 percentiles, and the plus sign designates the means

CSF YKL-40 concentrations were significantly higher in patients with HAD, as compared to all other groups (Fig. 1 and Tables 1 and 2). NA patients in all CD4+ T cell count strata < 350 cells/μL had significantly higher levels of CSF YKL-40 compared to HIV-negative controls, while CSF YKL-40 in NA patients with CD4+ T cell count > 350 did not differ significantly from controls.

**Table 2:**
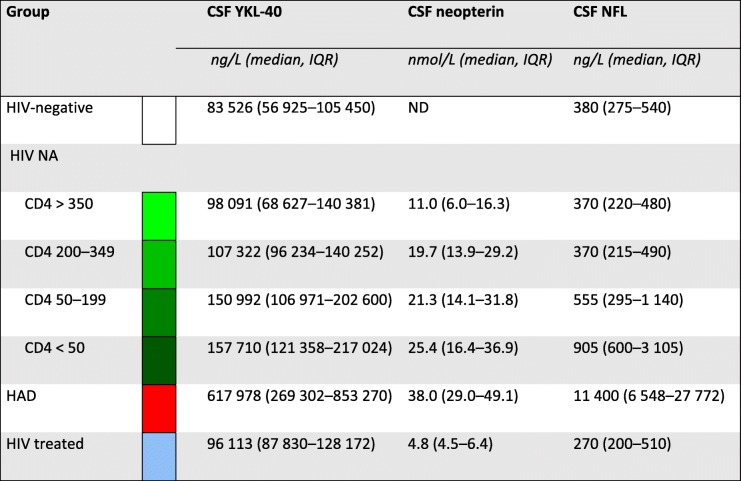
Median cerebrospinal fluid biomarker levels for the different groups of participants

*CSF* cerebrospinal fluid, *IQR* interquartile range, *ND* no data

**Fig. 2:**
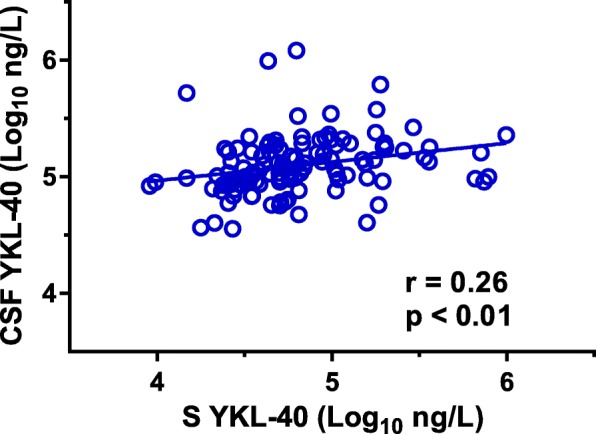
Correlation between serum and CSF (cerebrospinal fluid) YKL-40 in 115 HIV-positive patients and 5 controls, using the Pearson correlation coefficient

Serum YKL-40 was available for 120 individuals: 5 were HAD patients, 82 were NA, 28 were on ART, and 5 were controls. The mean serum levels of YKL-40 were 27% lower than CSF levels. There was a weak correlation between serum and CSF YKL-40 (*r* = 0.26, *p* < 0.01), but serum YKL-40 was not found to be a significant predictor of CSF YKL-40 or NFL in multivariable analyses (Fig. 2 and Table 3).

**Table 3:** Comparing group levels of CSF YKL-40, neopterin, and NFL using Tukey’s multiple comparison test after one-way ANOVA

Group comparisons	CSF YKL-40 (log)	CSF neopterin (log)	CSF NFL (log)
	Overall ANOVA *p*		
	< 0.0001	< 0.0001	< 0.0001
Tukey’s multiple comparison			
HAD vs. HIV–	< 0.0001	ND	< 0.0001
HAD vs. ART	< 0.0001	< 0.0001	< 0.0001
HAD vs. NA CD4 ≥ 350	< 0.0001	< 0.0001	< 0.0001
HAD vs. NA CD4 200–349	< 0.0001	ns	< 0.0001
HAD vs. NA CD4 50–199	< 0.0001	ns	< 0.0001
HAD vs. NA CD4 < 50	< 0.0001	ns	< 0.0001
NA CD4 < 50 vs. HIV–	< 0.0001	ND	< 0.0001
NA CD4 < 50 vs. ART	< 0.05	< 0.0001	< 0.0001
NA CD4 < 50 vs. NA CD4 ≥ 350	< 0.05	< 0.0001	< 0.0001
NA CD4 < 50 vs. NA CD4 200–349	ns	ns	< 0.0001
NA CD4 < 50 vs. NA CD4 50–199	ns	ns	ns
NA CD4 50–199 vs. HIV–	< 0.0001	ND	ns
NA CD4 50–199 vs. ART	ns	< 0.0001	< 0.05
NA CD4 50–199 vs. NA CD4 ≥ 350	ns	< 0.01	ns
NA CD4 50–199 vs. NA CD4 200–349	ns	ns	ns
NA CD4 200–349 vs. HIV–	< 0.05	ND	ns
NA CD4 200–349 vs. ART	ns	< 0.0001	ns
NA CD4 200–349 vs. NA CD4 ≥ 350	ns	< 0.05	ns
NA CD4 ≥ 350 vs. HIV–	ns	ND	ns
NA CD4 ≥ 350 vs. ART	ns	< 0.01	ns
ART vs. HIV–	ns	ND	ns

Median cerebrospinal (CSF) levels of YKL-40, neopterin, and NFL for the different groups

*IQR* interquartile range, *ND* no data, *ns* not significant, *NA* neuroasymptomatic, *HAD* HIV-associated dementia, *ART* antiretroviral therapy

**Fig. 3:**
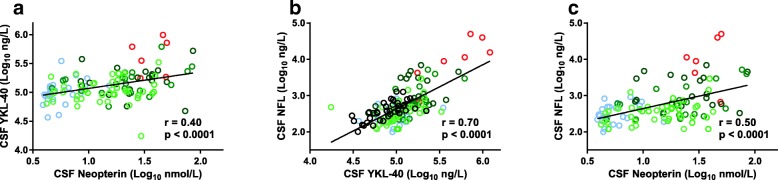
Correlations between CSF concentrations of **a** YKL-40 and neurofilament light protein (NFL), **b** neopterin and NFL, and **c** neopterin and YKL-40, using the Pearson correlation coefficient

When analyzing PLHIV with and without ART, we found significant correlations between CSF YKL-40 and age, CD4+ T cell count, CSF HIV RNA, CSF neopterin, albumin ratio, and CSF NFL (Table 4 and Fig. 3).

**Table 4:** Predicting cerebrospinal fluid YKL-40

Predictor	Univariable		Multivariable	
	Std b (*r*)	*p*	Std b_adj_	*p*
Age	0.378	< 0.001	0.418	< 0.001
CD4+ T cell count	− 0.380	< 0.001	− 0.285	0.002
Plasma HIV RNA	0.349	< 0.001		
CSF HIV RNA	0.350	< 0.001	0.298	0.005
Serum neopterin	0.287	0.002		
CSF neopterin	0.401	< 0.001		
Albumin ratio	0.435	< 0.001	0.223	0.004
Serum YKL-40	0.227	0.015		

Univariable correlation (left columns) and multiple linear regression (right columns) determining predictors of log_10_ CSF YKL-40 in 120 HIV-infected patients

*CSF* cerebrospinal fluid

When analyzing only people with effective ART, CSF YKL-40 correlated significantly with age (*r* = 0.58, *p* = 0.001), CSF neopterin (*r* = 0.38, *p* < 0.05), and CSF NFL (*r* = 0.375, *p* < 0.05).

In untreated PLHIV, including both NA individuals and patients with HAD, a significant correlation was found between CSF YKL-40 and age (*r* = 0.41, *p* < 0.001), CSF neopterin (*r* = 0.34, *p* = 0.001), albumin ratio (*r* = 0.45, *p* < 0.001), and CSF NFL (*r* = 0.73, *p* < 0.001). There was a significant negative correlation between YKL-40 and CD4+ T cell count (*r* = − 0.36, *p* < 0.001).

In controls, we found a significant correlation between CSF YKL-40 and age (*r* = 0.60, *p* < 0.001), albumin ratio (*r* = 0.43, *p* < 0.05), and CSF NFL (*r* = 0.68, *p* < 0.001). CD4+ T cell count and neopterin were not analyzed in the controls.

Age, CD4+ T cell count, albumin ratio, and CSF HIV RNA stood out as independent predictors of CSF YKL-40 concentrations in a multivariable analysis, including participants from all HIV groups (Table 4).

In addition, CSF YKL-40 was shown to be a strong independent predictor of CSF NFL in a multivariable analysis, together with age, CSF neopterin, and CD4+ T cell count (Table 5).

**Table 5:** Predicting cerebrospinal fluid NFL

Predictor	Univariable		Multivariable	
	Std b (*r*)	*p*	Std b_adj_	*p*
Age	0.402	< 0.001	0.203	0.003
CD4+ T cell count	− 0.530	< 0.001	− 0.259	< 0.001
CSF HIV RNA	0.348	< 0.001		
CSF neopterin	0.498	< 0.001	0.178	0.014
Albumin ratio	0.409	< 0.001		
CSF YKL-40	0.710	< 0.001	0.444	< 0.001

Univariable correlation (left columns) and multiple linear regression (right columns) determining predictors of log_10_ CSF NFL in 120 HIV-infected patients. CSF: cerebrospinal fluid

## Discussion

Astrocytes are considered important in the pathogenesis of HAD. Chronic intrathecal immune activation leads to impairment of the astrocytes’ physiological and neuronal support functions, and also to activation and proliferation of astrocytes [4]. We found that CSF levels of YKL-40, a marker of astrocytic activation, are significantly elevated in patients with HAD, compared to individuals on suppressive ART and to untreated NA individuals, irrespective of CD4+ T cell count. NA individuals with low CD4+ T cell counts (< 350 cells/μL) had significantly higher CSF YKL-40 compared to HIV-negative controls, but individuals with a preserved immune system (CD4 > 350 cells/μL) had CSF YKL-40 in the same range as controls. These findings support the conclusion that the activation of astrocytes is involved in HIV neuropathogenesis and in the development of HAD, as previously proposed by others [4, 5, 33].

One of our main findings is the strong correlation between CSF YKL-40 and CSF NFL, a sensitive marker of axonal injury [34, 35] indicating that there is an association between astrocytic activation and axonal injury. This is in agreement with a previous study with a correlation between CSF levels of YKL-40 and NFL in patients with chronic untreated HIV [29]. Correlations between YKL-40 and NFL have also been seen in MS [36]. In addition, we found albumin ratio to be an independent predictor of YKL-40. BBB disruption is mainly found in individuals with HAD and is thought of as a critical event in HIV CNS infection. Previous studies have also found that CSF neopterin and albumin ratio are independent predictors of CSF NFL, pointing towards a complex interplay between the activation of cells in the CNS, dysfunction of the BBB, and axonal injury in the pathogenesis of HIV in the CNS [37]. This further supports earlier findings suggesting that astrocyte activation is a major factor in this interaction and in the neuropathogenesis of HAD.

A similar pattern to that which we found for CSF YKL-40 has previously been reported for CSF neopterin during HIV infection [14]. Neopterin in CSF is probably the most studied marker of monocytic activation in HIV CNS infection. It is mainly produced by macrophages and microglial cells, but it can also be made by astrocytes, after stimulation with IFN-γ [38, 39]. Consequently, increased CSF neopterin concentrations probably reflect a multimodal activation of several different cell types. CSF neopterin levels are elevated early in HIV infection and remain elevated during all stages of infection, with the highest levels in individuals with HAD and CNS opportunistic infections. After initiation of ART, CSF neopterin decreases markedly but remains slightly abnormal in a large number of patients, despite several years of receiving ART [15].

CSF YKL-40 differs from neopterin in several ways. Neopterin is a small (253 Da) pteridine and YKL-40 is a 40 kDa protein. Although there is a possible overlap, as already mentioned, they largely represent the activation of different cell types. These are mainly macrophages and microglia for neopterin and astrocytes for YKL-40. We found a significant correlation between CSF YKL-40 and CSF neopterin among all HIV-infected individuals, whether or not they had neurocognitive impairment, or were or were not on ART. In a previous study [29], CSF YKL-40 correlated with neopterin in chronic, but not in acute HIV infection. The fact that the two biomarkers have different origins, or perhaps different mechanisms or time points leading to activation of the cellular sources, might explain some of the results in both of the studies. Although CSF YKL-40 does not have much clinical utility when compared to CSF neopterin, it is important to study various biomarkers that reflect different inflammatory pathways and activation of different cell types.

Soluble triggering receptor expressed on myeloid cells 2 (sTREM2) is a receptor glycoprotein belonging to the immunoglobulin superfamily. It is more specific for activation of microglia and macrophages than neopterin, since the secreted form of the receptor is exclusively expressed on myeloid cells such as macrophages and microglia, but not on astrocytes. It has become an established CSF biomarker selective for microglial activation and studied mainly in Alzheimer’s disease. In HIV infection, CSF concentrations of sTREM2 have been shown to be highest in patients with HAD and to increase in NA untreated individuals as the immune deficiency progresses [40].The same study also found a strong correlation between CSF sTREM2 concentrations and CSF NFL. It would be of interest to also study other inflammatory biomarkers and their association with YKL-40 and NFL. In a recent meta-analysis, CSF TGF-ß, MCP-1, and YKL-40 levels were significantly elevated in patients with neurodegenerative diseases such as Alzheimer’s disease, Parkinson’s disease, and amyotrophic lateral sclerosis [41].

Other markers of astrocytic activation have been suggested for studies in HIV. Glial fibrillary acidic protein (GFAP) is a major structural protein of astrocytes. Like YKL-40, CSF levels of GFAP increase in conditions with astrogliosis, such as Alzheimer’s disease and MS. There was no difference in CSF GFAP concentrations in individuals with HAD compared with individuals who had opportunistic CNS infections, HIV-infected NA individuals, or HIV-negative controls [42, 43]. Another astrocyte marker is S100β, an acidic calcium-binding protein that is abundant in astrocytes. CSF levels of S100β have previously been shown to be significantly higher in patients with moderate to severe HAD (formerly called AIDS dementia complex stages 2–3) than in patients with mild (stage 1) or no dementia. In addition, CSF S100β levels were higher in individuals with moderate to severe dementia who succumbed to their disease more rapidly than those who deteriorated more slowly with the same dementia stage [44]. It has not been shown to be useful in distinguishing HAD patients from patients with other neurological conditions [45], nor has it, to the best of our knowledge, been investigated in relation to NFL.

YKL-40 found in CSF is most likely produced in the CNS rather than in the periphery. We found a significant but weak correlation between CSF and serum YKL-40 concentrations. Serum levels of YKL-40 were 27% lower than CSF levels and were not shown to predict CSF YKL-40 in multivariable analysis. A similar lack of independent association between serum and CSF YKL-40 has previously been demonstrated in Alzheimer’s disease [22].

This study has several limitations. While the overall number of subjects was relatively large, individual groups were limited in size. The important HAD group was especially restricted by low incidence and availability making it difficult to draw conclusions about this group. Neurocognitive performance testing was not consistently done in non-HAD patients, making it impossible to evaluate CSF YKL-40 in patients with milder forms of HAND, such as asymptomatic neurocognitive impairment and mild neurocognitive disorder. CSF NFL has previously been suggested as a predictive marker of HAD [46]. The present study, however, has a retrospective cross-sectional design. A longitudinal study of CSF YKL-40 in HIV could further clarify its dynamics before and after the initiation of ART and perhaps resolve the efficacy of using YKL-40 as a prognostic marker or a marker of treatment response as well. Moreover, at a time where more and more PLHIV are on effective ART, studying a larger cohort on ART and patients with milder forms of HAND would be of interest to determine whether YKL-40 remains a useful marker that correlates with axonal injury in patients on ART. Controls were healthy volunteers, not matched with respect to demographic and lifestyle factors which has to be taken into consideration when interpreting the results [47].

## Conclusions

The focus of this study was to evaluate CSF YKL-40 in HIV-infected individuals with different stages of disease and with and without ART, and our results conclude that CSF YKL-40 is a promising candidate biomarker for understanding the pathogenesis of HIV in the CNS. The strong correlation between CSF YKL-40 and NFL suggests a pathogenetic association between astroglial activation and axonal injury and implies utility to evaluate the value of YKL-40 as a prognostic marker or marker of treatment response in HIV disease. It would also be of interest to study CSF YKL-40 longitudinally in individuals on ART with and without HAND.

## Abbreviations

ART: Antiretroviral treatment
BBB: Blood-brain barrier
CNS: Central nervous system
CSF: Cerebrospinal fluid
GFAP: Glial fibrillary acidic protein
HAD: HIV-associated dementia
HAND: HIV-associated neurocognitive disorder
HIV: Human immunodeficiency virus
IFN-γ: Interferon gamma
NA: Neuroasymptomatic
NFL: Neurofilament light protein
PLHIV: People living with HIV
SIV: Simian immunodeficiency virus
TNFα: Tumor necrosis factor alpha
